# Rethinking global digital health and AI-for-health innovation challenges

**DOI:** 10.1371/journal.pgph.0001844

**Published:** 2023-04-28

**Authors:** Andrew Farlow, Alexander Hoffmann, Girmaw Abebe Tadesse, Deogratias Mzurikwao, Rob Beyer, Darlington Akogo, Eva Weicken, Tafadzwa Matika, MaryJane Ijeoma Nweje, Watu Wamae, Sako Arts, Thomas Wiegand, Colin Bennett, Maha R. Farhat, Matthias I. Gröschel

**Affiliations:** 1 Oxford Martin School, University of Oxford, Oxford, United Kingdom; 2 Nuffield Department of Medicine, University of Oxford, Oxford, United Kingdom; 3 Department of Biomedical Informatics, Harvard Medical School, Boston, MA, United States of America; 4 IBM Research–Africa, Nairobi, Kenya; 5 Villgro Africa, Nairobi, Kenya; 6 MinoHealth AI Labs, Adenta Municipality, Ghana; 7 Fraunhofer Heinrich Hertz Institute, Berlin, Germany; 8 Clinton Health Access Initiative, Boston, MA, United States of America; 9 International Telecommunication Union, Geneva, Switzerland; 10 FruitPunch AI, Eindhoven, Netherlands; 11 Technical University of Berlin, Berlin, Germany; 12 Division of Pulmonary and Critical Care, Massachusetts General Hospital, Boston, MA, United States of America; 13 Department of Infectious Diseases and Respiratory Medicine, Charité –Universitätsmedizin Berlin, Berlin, Germany; Indian School of Business, INDIA

## Abstract

Digital health technologies can help tackle challenges in global public health. Digital and AI-for-Health Challenges, controlled events whose goal is to generate solutions to a given problem in a defined period of time, are one way of catalysing innovation. This article proposes an expanded investment framework for Global Health AI and digitalhealth Innovation that goes beyond traditional factors such as return on investment. Instead, we propose non monetary and non GDP metrics, such as Disability Adjusted Life Years or achievement of universal health coverage. Furthermore, we suggest a venture building approach around global health, which includes filtering of participants to reduce opportunity cost, close integration of implementation scientists and an incubator for the long-term development of ideas resulting from the challenge. Finally, we emphasize the need to strengthen human capital across a range of areas in local innovation, implementation-science, and in health services.

## Introduction

Artificial intelligence (AI) and digital health technologies are expanding rapidly and have the potential to transform health of populations across high-resource and low-resource settings and everywhere in between. Mobile applications and data-driven AI can help to tackle major challenges in global health. These include provision of health care during a pandemic [1], the wider sharing of accurate health knowledge and its use to serve public health goals [2], and the more equitable delivery of health care and health innovations [3, 4]. Despite their promise, AI and digital health technologies may also reinforce inequities in access to, and distribution of, health care services. LMICs have often faced delays in access to drugs and vaccines. If AI and digital health tools are developed to prioritise the needs of the wealthy over the needs of the poor–including in LMICs where very profitable sections of the population exist alongside many more who are poor–this pattern will repeat [5]. Meanwhile, a distinguishing feature of digital health tools is their ability to work in low-resource settings; indeed, low-resources can be a spur to innovation if there is the right support and investment model in place.

However, the process of translating advances in digital health and AI-for-health technologies into sustainable scaled-up health applications in resource-limited settings is complex, risky, and poorly understood. Innovation in digital health and AI-for-health works differently to innovation in drugs, vaccines, and diagnostics development. There have been successful AI-for-Health diagnostics that are in use in resource rich health systems [6–8]. Developing drugs or vaccines involves large, resource-intensive, randomised controlled clinical trials of essentially homogenous products. Decision-making on global vaccine research programmes and allocation of resources is made by research funders, foundations, committees of the WHO, GAVI, UNICEF, and others; local populations are involved in the sense that they participate in trials. The driving force behind digital and AI innovation and impact in LMICs is much more in the hands of local populations, and the design and use of digital tools depends much more on local contexts and even more on the other interventions (including other digital tools) that new digital tools will need to work with. In the case of drugs, vaccines, or diagnostics knowledge is embedded in products, diffused by sales; in the case of digital health and AI-for-health, knowledge diffusion is variable by context, and ‘use’ and ‘innovation’ go much more hand-in-hand. To fully reach their potential, we need a different kind of innovation model for digital health and AI-for-health in low-resource settings, one that is much more tailored from the start to local innovators and local intelligence, with allocation of resources driven by LMICs themselves, and especially by the poorest two billions of the world’s population. But how might this be done?

Digital and AI-for-Health Challenges (‘Challenges’)–controlled events whose goal is to generate solutions to a given problem in a defined period of time–are one approach to catalysing innovation that is sensitive to local context and focused on these populations [9–11]. There is a burgeoning amount of digital and AI innovation in LMICs. Challenge events are not intended to focus on most of this activity but to create opportunities to focus some of it on global health priorities and the needs of the poor when otherwise innovation would bypass them, to identify, test, and iteratively feed lessons learned back to improve design, and accelerate and sustain the scale-up of impactful quality solutions in low-resource settings. Such Challenges create opportunities to de-risk the development process of new global health technologies, by enabling the global health community to explore potential global health solutions at an early stage, to fail quickly, fail cheaply, and move on. Such risks might otherwise hold back development and deployment in resource-poor settings where there are not typical pre-seed-stage investors to absorb such risks.

The WHO and many others are expanding their digital health and AI-for-health initiatives and becoming increasingly aware of the ubiquitous potential, but also risks, of digital and AI tools in every area of health and at all levels of society and especially amongst those with the least resources of all. Whether a proposed AI or digital health innovation is a good innovation in a particular low-resource setting is best understood by those with local knowledge. We argue that therefore a ground-up, locally-informed, locally-led, approach to innovating and applying digital health and AI-for-health solutions–as a complement to top-down decision-making processes and funding mechanisms–should be key to any such global initiative, and that problem-focused Challenge events can be an effective way to draw out those with local knowledge and inspire them to bring about innovation in Global Health tools. If so, we need to refine and improve the Challenge concept to make it fit for this purpose. Here we highlight several recommended changes to the way future Challenges are conducted, assessed, and implemented including impact metrics that make more sense for global health and for the specific contexts in question, and we propose a supporting ‘Venture’ structure to sustain solutions.

## The current ‘Return on Investment’ paradigm is unsuited for digital health and AI-for-Health Innovation in the lowest of resource settings

Many pitches in typical tech hackathons and tech Challenges are based on the potential for high returns on initial equity investments based on expected profits in the marketplace [12]. This often does not align well with the most critical global health needs, as there is little profit to be made from many high-impact global health interventions. For example, if a test for an infectious disease already is low cost (e.g., 10 cents per test), it is difficult to earn even a small profit per purchase. There also tends to be a bias towards rewarding headline-seeking software innovators and ignoring the risks and needs of innovators of the less interesting hardware components which they make and that take much longer to get to market and to yield profits. Attracting investors is harder still if pay-out of innovations comes in the shape of global public goods or hard-to-value–or even not valued–health outcomes such as prevention of ill health, or if several risky projects need to coordinate to achieve big life-saving results. Many of the drivers of good (and bad) health are public goods (or bads) or positive or negative externalities and emerge out of environmental and social processes. Conventional competitive market mechanisms don’t easily reward innovation to handle such phenomena.

Some global health initiatives have responded to the lack of profit derivable from low-resource settings by creating subsidies to top-up the prices to sellers (or to drive prices down for buyers) to make markets more attractive, but the process has always been much more complicated to enact than a simple parse of the problem might suggest [13]. Implementing top-down centrally-determined subsidies for a myriad of bottom-up context-adapted digital health tools would be even more practically challenging. In consequence, many digital health innovations to tackle global health challenges will need to rely on only the resources available in low-resource settings and will only occasionally benefit from external investments. Resources that might otherwise have gone into subsidising digital health and AI-for-Health can go into supporting those who succeed in Challenge events instead.

Typical venture capital investors usually seek more immediate returns on their investments and are less interested in the long-term strategy after they have cashed out their investments. The danger that deep-pocketed global corporations will come to dominate the digital-health scene in LMICs (as they are increasingly doing with mobile-phone technology and the internet), is further motivation for a mechanism that puts local entrepreneurs at the heart of decision making (while not ruling out the engagement of such corporations on social and economic ethical terms including driven by LMIC innovators on a more level playing field).

If not carefully designed, Challenges can create large opportunity costs to those who participate: Organizing a three-day Challenge with three thousand applicants equates to over 25 years of collective time taken away in resource-poor settings where the alternative use of that time (the true opportunity cost) is extremely high. It is unclear what all that effort of participants achieves or what the status of the solutions several years down the line will be. Recent studies on the sustainability of hackathon events, including large-scale quantitative studies, revealed that only 5–7% had any activity six months after the hackathon ended and that, in particular, intensive short-term activity in an effort to achieve a win in a hackathon is associated with lower likelihood of long-term project continuation [14, 15]; frenetic PR effort are not synonymous with long-term impact. Fewer, higher-quality, more targeted projects would be a better use of limited resources in low-resource settings. To reduce these transactions costs, several filters can be applied to narrow the field down in advance of any major investment of applicants in Challenge events. These filters–transparently displayed–include criteria on potential impact on global health, how scalable and implementable a potential solution is, and the nature of the support structure available to sustain solutions (perhaps with some funds provided to those successful in a Challenge event). Filters of participating teams should also ensure that partners and local stakeholders–including governments who will often end up financially implicated–are involved early on.

## An expanded investment framework for global health AI and digital health innovation

Much digital health innovation is driven by the potential for profit in high-income economies. While not excluding this as a motivation for innovation, we propose an investment model based on expected global health returns, such as Disability Adjusted Life Years (DALYs) prevented over future horizons, and wider health and development impact. Increasingly, evidence on this is being generated by large global studies such as the Global Burden of Disease and by local-level surveillance (which is improving on the back of efforts to prevent future epidemics and pandemics) and by the surveillance and data-gathering of health-systems themselves, enabling global health returns to be more geographically determined and related to local health burden [16, 17]. Part of a possible investment metric might be the contribution of digital health technologies to achieving Universal Health Coverage (UHC), proposed by the World Bank and WHO as “everyone–irrespective of their ability-to-pay–gets the health services they need in a timely fashion without suffering any undue financial hardship as a result of receiving the care” [18].

It has long been known–but only recently become much more prominent in global health thinking–that human health is shaped by the natural environment, planetary health, climate change, and biodiversity under the One Health mantle [19], and by broader societal and economic forces which affect accessibility and equity. The WHO, in the concept of ‘Health for All’ puts forward a model for governance and finance to harmonize and sustain benefits from innovations in health care, in addition to those from regular commercial activity or charitable efforts [20]. Thus, any long-term vision of health at the heart of digital health and AI-for-health technology challenges has to be holistic with a concomitant broad notion of health technology innovation to capture systemic planetary health aspects.

Challenges in Global Health can also be part of the broader endeavour to create new non-Gross Domestic Product (GDP) measures of value and investor return. GDP puts too little value on public goods and positive externalities including in global public health, such as prevention of poor health outcomes and epidemic and pandemic prevention, and impacts–good and bad–on natural capital and on human capital. Moreover, low prices–driven by low income and lack of affordability amongst the poor–manifest as low GDP value placed on interventions even when the true value in terms of better health and lives saved might be high.

**Fig 1:**
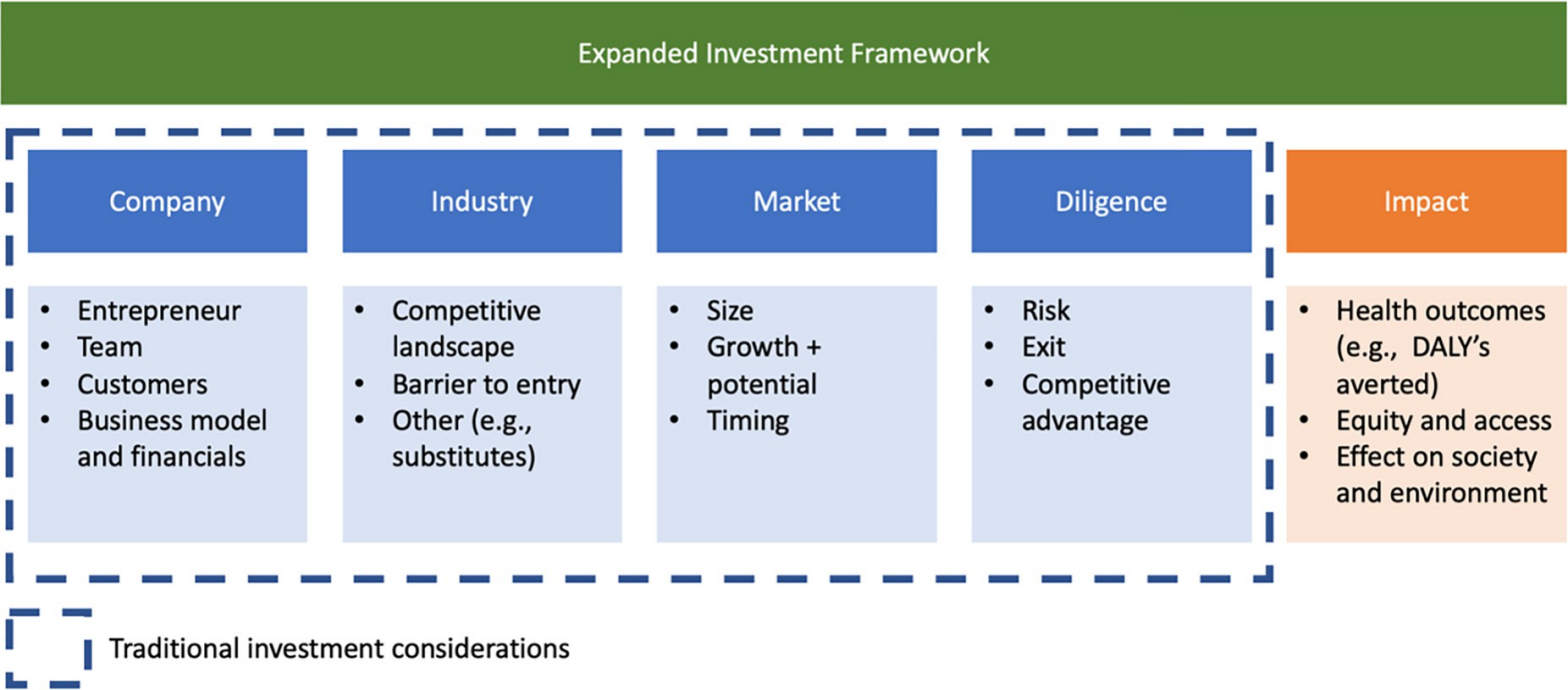
Expanded investment framework. This expanded investment framework is a tailored form of impact investing that goes beyond traditional factors (e.g., profit) to include health outcomes among other outcomes (such as impact on the environment). The traditional investment framework is representative but not exhaustive and demonstrates many of the factors that venture capital and private equity investors typically consider when investing in digital tools. Each specific investor may prioritize different aspects of this framework and may even consider other factors too. It is likely that publicly backed funds/research funding consider very different criteria as well. The traditional investment model is adapted from references [35, 36].

How might we identify and reward some of this ‘hidden’ non-monetizable value created by digital health and AI-for-Health innovation? The solution is to expand the investment framework used to evaluate Challenges (Fig 1) to more closely incorporate the true ‘shadow prices’ of global health digital and AI technologies in terms of their DALY impact and lives saved, borrowing techniques from groups developing shadow prices for activities that impact the environment such as the Dasgupta Review on the Economics of Biodiversity [21].

A shadow price is an estimated price for something that is not normally priced or sold in a market (such as mangroves that are extremely valuable at stabilizing coastlines against erosion and enhancing biodiversity, or rainforests that create value by absorbing carbon and so help to tackle climate change) or that is sold in a market but the market price of which does not capture the full benefit (such as new electric transport technologies at least part of the return on which is their positive impact on climate change and health). A shadow price gives a better understanding of the benefits. When the buying power amongst populations, for example in LMICs, is weak or non-existent, we especially need a way to find better value health interventions in those populations, and we hypothesize that DALYs, shadow prices, and concepts such as the value of insuring and protecting the health of the poor, give us a framework to do this [22, 23]. Digital health and AI-for-Health are proliferating at an opportune moment to take advantage of new global economic accounting rules being developed to better incorporate these different sources of value (as alternatives or supplements to GDP accounting rules) [24].

Adding more impact measurements to Challenge events may suggest that innovations that are impactful but difficult to measure by these new metrics might fall by the wayside. However, creating new metrics will prompt organizers of Challenge events to innovate in how they measure impact in less conventional senses such that these alternative metrics improve over time. Further, shadow prices and other approaches might improve enough, by their more regular application to health, to be able eventually to measure some difficult-to-measure impacts. Third, if impact measurements have a high degree of uncertainty, sensitivity and scenarios analyses could be performed to quantify the impact of this uncertainty. No impact metrics are perfect including current ones, and we should not argue for perfection; the hope is that if an innovation has great impact even if it is hard to measure, these metrics will increase the chances of that innovation being funded and scaled up.

We recognise that measuring impact can be complex and costly and may divert funds from hosting events or scaling up innovations. The increasing importance of quantitative evidence can lead to a situation in which “only those operations which are counted and can be counted, count at all, and that qualitative and more complex operations will receive less and less attention” [25, 26]. To generate sustainable impact, Challenge events need to be practically meaningful, even if this means that fewer events can be run.

## An expanded data framework for global health digital health and AI-for-Health innovation

To successfully repurpose and use data for digital health innovation at larger scales requires a mutual and equitable understanding of data sharing. Investments are needed in three areas: First, in health monitoring data to increase the amount of data available to make Challenge events attractive to participants. Improved health information systems in low-resource settings are also critical to effective local implementation, management, and performance measurement. Second, in strengthened equitable data-sharing infrastructures to underly the evaluation and implementation of solutions (to include how data is stored, shared, protected, and the role of open source). Bottom-up innovation is dependent on the availability of data to local on-the-ground innovators who understand the overall context. Increased data equity incentivizes effective private and local innovation; without data for AI models to learn from, there can be no AI. This will necessitate counterbalancing the power and resources of the likes of Microsoft, Google, Facebook, AXA, and their equivalents with local voices and resources, and integrating equitable data-sharing protocols into all areas. Third, in improved health technology assessment and cost effectiveness/economic evaluation frameworks, which are rendered even more complicated by the complex nature of most packages of interventions and the way some tools are public goods (with large sunk costs and very low, and even zero, marginal costs). The ITU-WHO is currently working on strengthening capacity to do this analysis.

## Implementation and sustainability are key

A piece of “naked digital health technology” is not on its own a solution. Turning it into high-impact global health interventions in resource-limited settings is not easy. There exist hundreds of thousands of failed digital apps with a high risk of the bad driving out the good [27]. This makes it risky for innovators of high-quality innovations. Implementation challenges differ radically across settings, with especially big differences between health systems that are publicly funded and those that are privately funded. And there is always the danger that implementation will drift towards inequity. Implementation science, so often an afterthought, should be at the heart of any digital health technology innovation developed during a Challenge event. Otherwise, there is a risk that new technologies will not fit health-delivery workflows nor help those providing or receiving care in their own day-to-day activities. Many innovations have no value to end users even if they have a logical value for developers. Indeed, innovations can impress by not being exposed to end users too rigorously, too early. It is better not to encourage innovators to waste their time on those technologies but instead focus their time on innovations more likely to work in real-world settings; the conducting of Challenges must built this logic in from its start. Furthermore, perspectives shaped by academic approaches may not fully reflect the local context and on-the-ground realities, which are often much better understood by practitioners and innovators with years of experience in local settings and human-centered design [28]. Also, diversity in both academic and non-academic personnel is crucial to scaling up digital solutions that adapt to the local context; bringing these groups together and incorporating local knowledge into the design of digital health technology innovations and the way Challenges are conducted, proffers multidirectional benefits.

The implementation of some solutions might benefit from a “Theory of Change” drawn up with the help of all stakeholders. In nature conservation, frameworks such as Foundations of Success [29] (FOS) describe the steps needed to better understand the results of interventions on the entire system under consideration, defining objectives, goals and indicators for each given step, and Outcome Action Plans (OAPs) that utilize adaptive management to plan for, measure, and improve on projects in action. In addition to better outcomes, this allows for communication of results using a consistent framework and vocabulary, which builds trust among stakeholders. Similar processes adapted for the health care context have been shown to improve the chances of successful changes over time [30].

Beyond the ‘design’ stage, active participation of various stakeholders is essential in experimenting with, deploying, and monitoring digital solutions. Brilliant ideas don’t just happen; they require lots of testing, mistakes, and failures on the pathway to success. Particularly, a human-in-the-loop approach is beneficial to incorporate latest updates into digital health and AI-for-Health solutions via a feedback loop, to achieve long-term sustainable use. Policymakers, service providers, local innovators, and patients in routine settings become partners in a digital-health and AI-for-Health learning enterprise rather than just passive recipients or ‘consumers’. Their engagement, and their sense of control and ownership, increases the chance that solutions are appropriate and sustained after any initial development of a piece of tech or a ‘pilot’ stage. In such ways, common data standards and long-term interoperability can be shaped by the voices of patients and service providers and, critically, be matched to constraints in real-world settings, which is essential for accelerating applications in resource-poor health settings. Finally, much of the funding into analysing digital health technologies goes into academic research projects, largely limiting the sustainability of the projects when the funding ends. Linking academic research projects with innovators early on may result in more commercialisation of research findings. All of the above needs to be at the heart of mechanisms to shape Challenges events and not left till after such Challenges have ended.

## A venture building approach around global health innovation

**Fig 2:**
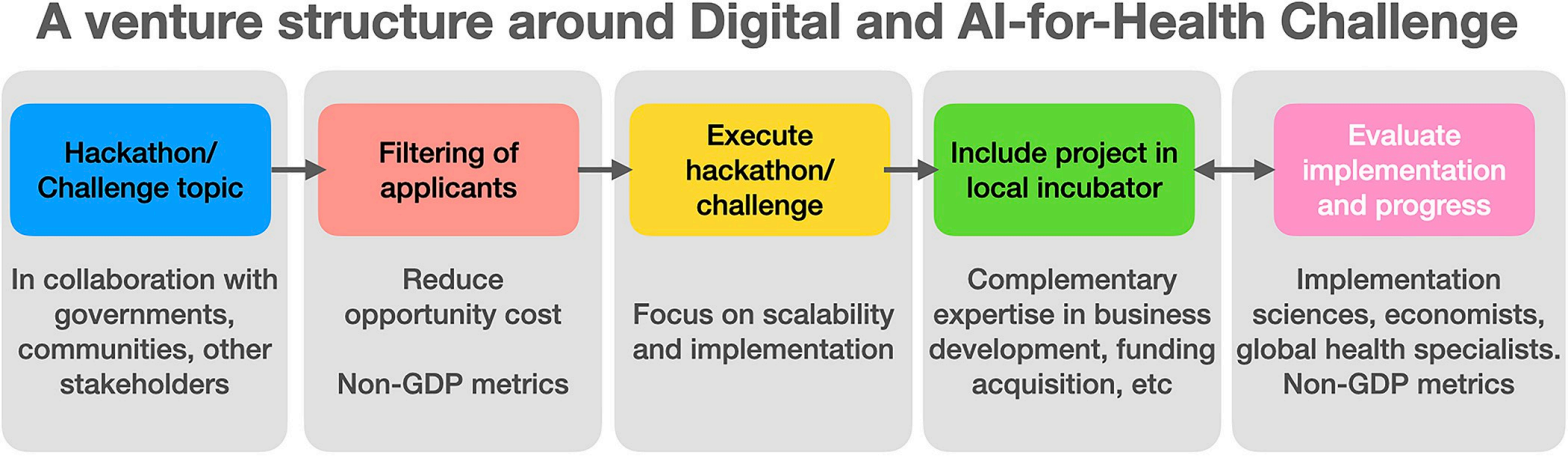
A venture building approach around challenges in global health. Any Challenge needs to be nested in a venture building approach guiding the development of digital health technologies. This includes filtering of participants, close integration of implementation scientists to ensure sustainability and scalability, and an incubator following the Challenge event for long-term development of the ideas resulting from the Challenge.

The practical experiences of many of those working in digital health innovation in low-resource settings recognise that innovation takes more than good ideas. To achieve impact, all the above processes need to be nested in a ‘venture building structure’, a supportive ecosystem able to incubate and support locally-driven digital health and AI innovators, i.e., the Challenge event all the way from idea to evidence to innovation to scaled-up implementation and long-term sustainability (to at least two-years post Challenge event), going beyond the typical short periods of many technology-based hackathons (Fig 2). This needs to engage many diverse partners–including those collecting data, academic institutes, government, private, and civil society actors.

We propose this new venture building approach as a form of organisational innovation that will apply less of a traditional business venture building lens but have its focus on the global health impacts described above. The final goal is that solutions stemming from Challenge events stand on their own feet, and are investable to funders including impact investors, and they successfully transition from academic projects to private entities to ensure sustainability and implementation.

Commercialisation of innovation coming out of such Challenges is not incompatible with global health and indeed, is a necessary part of a sustainable response. The key insight is to align commercial incentives with global public health priorities and to make sure that all non-commercial stakeholders (including funding bodies) leverage their resources to shape the commercial landscape around global public health priorities and that they do not passively accept extant commercial priorities.

By creating a consortium of interested parties with expertise across the entire ecosystem of support needed, deploying scientific rigor and due diligence, more high-quality interventions will make it into use, and the current sea of poor-quality innovations and waste can be reduced [27]. The venture structure will reassure that good interventions will more likely have uptake, and investment will be de-risked.

Investors with interests in green investments with payoffs articulated in terms of environmental impacts may equally be interested in investments with global health impacts. But exactly how this might be achieved is a work in progress, and experiences from other areas of environmental and biodiversity impact suggest that this remains a huge task.

## Invest in human capital

Evidence indicates that short-term continuation after winning most typical hackathons is enhanced by technical preparation and the number of technologies pulled together to achieve the win, but that long-term continuation is much more dependent on skill diversity amongst the team, their technical capabilities in relation to the technologies, and their intention to expand the project [11]. Human support to nurture new ideas can often be much more useful to start-ups than just money.

A ground-up design-driven process in LMICs will need to support local ‘intelligence’ to improve health data in LMIC hospitals in terms of skills and the organisation of people to meaningfully use such data, and health systems that are continuously learning and improving. This will need strategies to strengthen human capital across a range of areas in local innovation, implementation-science, and in health services. More broadly, attention is needed to the training of more mathematically astute bio-medics, bio-chemics, and medical/clinical specialists as well as integrating all of the above into ongoing research and teaching activities [31].

Challenges have previously been shown to be a useable tool in education [32]. In many LMICs, there are few universities offering AI related courses. Most AI researchers and practitioners attain their skills through online resources where there is limited ethical and tailored training [33]. If we are to lean heavily on local innovators and experts and not over-rely on external expert mechanisms, this might limit the development of sustainable AI solutions, which need to be explainable, ethical, secure, and human-centred (as discussed in the research field of Responsible or Trustworthy AI). Generally, developing trustworthy AI solutions requires detecting and mitigating systematic deviations/biases. Due to the limited training data and computational resources, AI practitioners in LMICs tend to utilise data (trained in different geographical locations and contexts) and models (designed and fine-tuned for different problem settings) that result in systemic biases towards the population in LMICs [34]. The Challenge agenda therefore needs to include training on evaluating data, and models for detection and mitigation of hidden errors and biases, and boosting capacity to do health technology assessment and cost effectiveness analysis.

In case of time constraints during Challenge events, some of these skills could also be developed by online courses offered to Challenge participants. Following the practices of some who already run or advise on challenge-style events (e.g., FruitPunch, Wadhwani AI, Villgro Africa, AI for Good), this can be supported by certificates and accreditation of those applying their skills for the social good so that Challenges can be used to invest in human capital.

When designing challenges, we need to think in terms of the incentives of participants who–because large prize money is not possible in resource-poor settings–mostly take part because of the opportunity to apply their skills for the common good of others, to improve their ability to apply AI in practice, and to experiment with state-of-the-art AI. For these participants, the real value is in unique datasets and problem statements that suggest possibility for significant impact; without interesting datasets, interesting problem statements alone are not enough to attract quality participation. Digital health and AI-for-Health Challenges offer chances for building a global community of practice around digital health and AI-for-health that could not be achieved in any other way.

The need to invest in human capital is not unique to digital health and AI-for-Health. Limited human capital is a barrier to addressing epidemics and global pandemics, to finding new antibiotics, and to discovering new medicines or treatments in general, including from evaluating more ancient, natural-product, pathways, and remedies. Investing in human capital–in this case relating to AI and digital techniques–is an oft overlooked prerequisite for success of many global health initiatives. Challenges and associated venture structures can be designed to strengthen human capital which has impact way after the Challenge and whether or not participants succeed in such Challenges. One example is the National Institutes of Health funded Data Science for Health Discovery and Innovation in Africa (DS-I Africa), which aims to enhance organizational and workforce capabilities in data science research, training, open-source tools, ethics, and legal frameworks in Africa, to enable research networks of local investigators to leverage data science technologies applied to health in their own settings.

## Conclusions and future direction

The global health outlook has been severely tested by the COVID-19 pandemic. The risk to public health of being inadequately prepared for future health crises is high. Adoption of new innovations in care is ever more urgent, yet the current model of digital health and AI-for-Health innovation is not optimized to address global public health priorities. With complex issues that affect many parties, productively working alone in a vacuum is not possible; decision-makers such as governments and healthcare businesses need to find ways to work together to develop trust and co-create value.

An open question remains who will ultimately pay for digital health innovations in low-resource settings. There is hope that some of the interventions create savings; ether because they reduce costs of medical treatment since once an improved algorithm or imaging software is identified, it can replace an alternative cheaply. Or they are preventative and take negative health consequences away. The problems such interventions face in seeking payment are the same as are faced by other more traditional interventions which can also struggle to demonstrate cost-saving value, except that now we hope to have more metrics to demonstrate any hidden value. Preventative tools notoriously go under-remunerated because the payers are poorly aligned with beneficiaries (an ex-post medical treatment is a cost that health services are happy to incur, but a preventative measure in the community will be unfunded by a health service). Governments, especially in low-resource settings, should be included early on to increase chances that they adopt and ultimately pay for digital health innovations, and because many of them are motivated by their goal to achieve Universal Health Coverage to seek out affordable innovations. We also visualize many innovations being affordable even amongst the poorest, given low marginal costs and the ubiquitous ability to exploit current technology.

While the Challenge format is a promising way to catalyse innovation in Global Health in low-resource settings, a set of guidelines needs to be created collectively as part of any new global health digital health or AI-for-health initiative. Current models looking for more immediate gains and return on investments need to be replaced with new models based on metrics such as DALYs, UHC, and shadow prices. Innovations developed within a Challenge need to be conceived with a focus on implementation to ensure solutions create maximum impact and benefit. To ensure any digital health technology is developed in a sustainable and scalable framework, a venture building approach is needed that includes all stakeholders, implementation scientists, and business development incubators. Challenges supported by such a venture-building approach could be one promising driver of Global Health innovation linking outcomes to needs as part of new global digital health and AI-for-health initiatives.
